# Do Human Capital Investment and Technological Innovation Have a Permanent Effect on Population Health? An Asymmetric Analysis of BRICS Economies

**DOI:** 10.3389/fpubh.2021.723557

**Published:** 2021-07-21

**Authors:** Gang-Gao Hu, Li-Peng Yao

**Affiliations:** ^1^Business School of Ningbo University, Ningbo, China; ^2^Ningbo College of Health Sciences, Ningbo, China

**Keywords:** BRICS, human capital, NARDL, population health, PMG panel, technological innovation

## Abstract

This study examines the asymmetric impact of human capital investment, and technological innovation on population health from the years spanning from 1991 to 2019, by using a panel of the BRICS countries. For this purpose, we have employed the PMG panel NARDL approach, which captures the long-run and short-run dynamics of the concerned variables. The empirical results show that human capital investment and technological innovation indeed happen to exert asymmetric effects on the dynamics of health in BRICS countries. Findings also reveal that increased human capital investment and technological innovation have positive effects on health, while the deceased human capital investment and technological innovation tend to have negative effects on population health in the long run. Based on these revelations, some policy recommendations have been proposed for BRICS economies.

## Introduction

In its true essence, the demand theory suggests that consumers are used to ranking different mixtures of goods and services that they typically purchase on the basis of their individual utility function. According to this theory, consumers purchase those combinations of goods and services that increase their utility to the highest level, by optimally utilizing their income. Therefore, it can be affirmed that this theory is suitable for predicting the consumers' demand for goods and services. However, the demand function in medical economics puts forth the argument that when consumers acquire medical treatment or services, they actually expect and subsequently demand better to good health conditions and not only the services that are being provided. According to Bentham (1), the feeling and emotion of pain was included as one of the fifteen “simple pleasures” in a person's utility function, as early as the year 1789.

Positive investment in education is one of the primary sources of development of human capital for any nation. Education is defined as human capital primarily because it develops the productive capacity and increases the skill level of the people (2, 3). Researchers have suggested that formal education promotes and modifies the general skills and abilities of the people, rather than focusing on a specific purpose. When a person attains formal education, this process helps them in acquiring the mind-set that makes them much psychologically stronger in tough situations, and helps them solve problems that come in their way in a more informed and calculated manner. Among these problems, some are related to productivity, which is the main disciplinary focus of proprietors and economists. Some of these problems are related to basic personal needs, and one such critical need is health and healthcare (2, 3).

Education is a primary source of developing the real expertise of an individual, their capabilities, and skills, and is known as the most basic proxy that can be considered to calculate human capital. Hence, theory suggests that an enhanced level of human capital will lead to better health conditions, mainly because the advancement in human capital will alter the lifestyle of humans by changing their habits, mind-sets, skills, abilities, training, etc. Moreover, along with the development and enhancement of education, people's realization about better health conditions also tends to increase. Furthermore, education not only helps to make people aware of different health issues, but also serves as a means through which better health standards can be achieved (4). Through superior training courses that are focused toward enhancing the knowledge and skills attained by doctors and medical staff, the general health conditions of people can be improved, and this can effectively be attained by investing heavily in health-related infrastructures.

The empirical literature available on the education-health nexus also suggests that education is the main driver of improved health conditions. In addition to this, education in various different fields of study, on one side, improves the physical working and individual health of the people of all ages and at the same time also helps to decrease the rate of sickness, incapacity, and death that are related to aging and other related factors (5–9). Moreover, studies pertaining to researchers such as Wilkinson and Spurlock (10), and Doornbos and Kromhout (11) actively argue that it is education that exerts a positive impact on health and healthcare related disciplines, and it is essentially not health that improves the level of education in people.

Among the other aspects of human capital that can be taken under consideration, the most significant ones pertain to the spending on research and development, and technological innovations (12). These two areas of study are increasingly becoming popular, especially in their role in collecting empirical evidence based on the impact of human capital, on all sectors of the economy. Investment in research and development has done wonders in emerging economies and is actively considered as a key driver of economic growth (13–17). Eventually, this increased investment in research and development will lead the nations toward technological innovations that can affect the economy through various channels. In this regard, we can dwell a little deeper into these effects. Firstly, it will improve the competitive position of the country in the global market and will lead to a boost in the exports of a country. Secondly, innovations will help countries to develop more advanced financial systems. Thirdly, the overall infrastructure of the countries will improve, and as a result, people will enjoy higher standards of life and living (18). Similarly, more spending on research and development will help in creating sophisticated infrastructure that can be directly proportional to the enhancement of health care services i.e., hospitals, testing laboratories, medical equipment, and medicines etc. In addition to this, investment in research and development initiatives provides the society with better trained and highly qualified doctors and paramedical staff, who will eventually prove to be catalysts in improving the health standards of the people that they treat (19, 20).

The investment in human capital (education, research and development, and technological innovations) can also affect the health status of people, albeit through an indirect channel i.e., environment. However, though, as the investment in human capital spurs economic activity, this can also harm the quality of the environment (21, 22), which will deteriorate the health status of the general public as well. Contrariwise, human capital can also improve the environmental quality of society through better and more sophisticated techniques of production, efficient use of energy, increased use of environmentally friendly products, and creating awareness about the greener and cleaner environment (23). Several studies show that a cleaner and greener environment certainly tends to have a positive impact on people's health—be it physical, emotional or psychological in nature (24, 25).

In this study, our basic goal is to analyze whether the investment in human capital actually matters when it comes to the health condition of the people belonging to BRICS (Brazil, Russia, India, China, South Africa) economies. It must be noted that the BRICS economies are among the fastest-growing economies of the world, and are home to about 3.2 billion people of the world. It must also be noticed that the collective GDP of these economies is about 20.81 trillion US$. Hence, it is safe to affirm that these economies are capable of serving as an ideal case study, in order to analyse the impact of investment in human capital, on the health status of the people. This study is more important because average life expectancy in BRICS stands a fraction below the average of global (26). The BRICS economies are facing a lot of challenges in achieving sustainable development goals.

Few empirical studies identifying the important factors of BRICS health outcomes, such as health spending (27, 28); health system (29); health care financing (30, 31); GDP (26); none of these studies examined the impact of human capital investment and technological innovation on BRICS health outcomes. Previous literature assumed the linear impact of health expenditure on health efficiency. These previous studies cannot assess the impact of technological and human capital shocks on health outcomes. For this purpose, we used the panel non-linear ARDL-PMG method of estimation for analysis. This methodology is easily captured the positive and negative shocks of technology and human capital investment on health outcomes.

To the best of our knowledge, this is the first-ever study that has included three different aspects of human capital, i.e., education expenditures, research and development expenditures (R&D), and technological innovations. Moreover, this study also aims to successfully perform a comparative analysis of these indicators on the health condition of the people living in BRICS countries. More importantly, the analysis is based on the non-linear ARDL model, which has the benefit of providing information regarding whether the health status of people in these economies responds to the variables of human capital symmetrically, or asymmetrically. Furthermore, the results of this study have not suffered from the problem of aggregation bias. The findings of this study are supportive for academicians, development practitioners, health institutions, and international organizations.

This study is comprised of various sections. Section two of the study writes the model and discusses the methodology of the paper in detail. Moreover, in section three, we have elaborated upon the results, while the study is concluded in the fourth section of the paper.

## Model and Methods

By analyzing the extant literature in-depth, in order to capture the impact of human capital on the life expectancy of the people living in the BRICS economies, we have borrowed the following model:

(1) $$\text{Life }{\text{expectancy}}_{\text{ it}}=\varphi_{0}+\varphi_{1}{\text{EE}}_{\text{it}}+\varphi_{2}{\text{TI}}_{\text{it}}+\varphi_{3}{\text{X}}_{\text{it}}+\varepsilon_{\text{it}}$$

In the above model (1), the life expectancy in BRICS economies depends on the education expenditure (EE), technological innovation (TI), and a set of control variables (GDP, education, internet) as denoted by the symbol X. The methodology adopted in this study is the linear and non-linear panel ARDL-PMG. However, the asymmetric version is the extension of the linear model, hence; we have initiated our discussion from the linear ARDL-PMG model. Therefore, the relationship described in Equation (1) will go on a temporal route before attaining a long-run equilibrium route. Hence, Equation (1) needs to be stated in the form of the ARDL model, as suggested by (32–34).

ΔLife expectancy_it_

(2) $$\begin{array}{l} =\;\omega_{0}+\;\sum_{k=1}^{n}\beta_{1k}\Delta \text{Life }{\text{expectancy}}_{\;i,t-k}\\ +\sum_{\text{k}=0}^{\text{n}}\beta_{2\text{k}}\Delta {\text{EE}}_{\text{i},\text{t}-\text{k}}+\sum_{\text{k}=0}^{\text{n}}\beta_{3\text{k}}\Delta {\text{TI}}_{\text{i},\text{t}-\text{k}}\\ +\;\sum_{\text{k}=0}^{\text{n}}\beta_{4\text{k}}\Delta {\text{X}}_{\text{i},\text{t}-\text{k}}+\omega_{1}\text{Life }{\text{expectancy}}_{\text{i},\text{t}-1}\\ +\omega_{2}{\text{EE}}_{\text{i},\text{t}-1}+\omega_{3}{\text{TI}}_{\text{i},\text{t}-1}+\omega_{4}{\text{X}}_{\text{i},\text{t}-1}+\varepsilon_{\text{t}} \end{array}$$

Equation (2) is formerly known as the ARDL model of (32–34). In this model, the estimates attached to first difference operator, i.e., Δ, represent the short-run results, whereas, the long-run results are signified by the coefficient estimates of ω_2_− ω_4_, normalized on ω_1_. In this regard, in order to prove that our long-run results are valid, we need to prove the co-integration among the long-run estimates. To that end, the error correction term (ECT_t−1_) has been developed by using the estimates from Equation (1), and the estimate of ECT_t−1_ should be negative and significant in nature. Moreover, most of the macroeconomic variables have become stationary after differencing only once, hence, the leading benefit of applying this methodology is that it capable of performing well, even if the variables are I(0), I(1), or a mixture of both.

The basic aim of this study is to observe the asymmetric impact of the variables of human capital, on the life expectancy in BRICS countries. Therefore, we needed to divide the variables of our interest i.e., education expenditure (EE) and technological innovation (TI), into their positive (${\text{EE}}_{\text{it}}^{+}$, $\text{T}{\text{I}}_{\text{it}}^{+}$) and negative (${\text{EE}}_{\text{it}}^{-}$, $\text{T}{\text{I}}_{\text{it}}^{-}$) parts, by using the partial sum technique as proposed by Shin et al. (35). This has been presented below as:

(3a) $$\begin{array}{l} {\text{EE}}_{\text{it}}^{+}=\sum_{\text{n}=1}^{\text{t}}\Delta {\text{EE}}^{+}{}_{\text{it}}=\sum_{\text{n}=1}^{\text{t}}\max\;(\Delta {\text{EE}}^{+}{}_{\text{it}},0) \end{array}$$

(3b) $$\begin{array}{l} {\text{EE}}^{-}{}_{it}=\;\sum_{\text{n}=1}^{\text{t}}\Delta {\text{EE}}^{-}{}_{\text{it}}=\sum_{\text{n}=1}^{\text{t}}\min\;(\Delta {\text{EE}}^{-}{}_{\text{it}},0) \end{array}$$

(3c) $$\begin{array}{l} {\text{TI}}^{+}{}_{it}=\;\sum_{\text{n}=1}^{\text{t}}\Delta {\text{TI}}^{+}{}_{\text{it}}=\sum_{\text{n}=1}^{\text{t}}\max\;(\Delta {\text{TI}}^{+}{}_{\text{it}},0) \end{array}$$

(3d) $$\begin{array}{l} {\text{TI}}^{-}{}_{it}=\;\sum_{\text{n}=1}^{\text{t}}\Delta {\text{TI}}^{-}{}_{\text{it}}=\sum_{\text{n}=1}^{\text{t}}\min\;(\Delta {\text{TI}}^{-}{}_{\text{it}},0) \end{array}$$

In the above equations the functions, $\text{E}{\text{E}}_{\text{it}}^{+}$ and $\text{T}{\text{I}}_{\text{it}}^{+}$, signify the positive changes or shocks, whereas, the functions $\text{E}{\text{E}}_{\text{it}}^{-}$ and $\text{T}{\text{I}}_{\text{it}}^{-}$ signify the negative changes or shocks. After breaking down the respective variables, the next step was to substitute these partial sum variables in the space of the original variables that were mentioned in Equation (2). When the substation takes place, the new equation tends to look like as shown below:

ΔLife expectancy_it_

(4) $$\begin{array}{r} =\alpha_{0}+\sum_{\text{k}=1}^{\text{n}}\beta_{1\text{k}}\Delta \text{Life }{\text{expectancy}}_{2,\text{it}-\text{k}}+\sum_{\text{k}=0}^{\text{n}}\beta_{2\text{k}}\Delta {\text{EE}}^{+}{}_{\text{it}-\text{k}}\\ +\;\sum_{\text{k}=0}^{\text{n}}\delta_{3\text{k}}\Delta {\text{EE}}^{-}{}_{\text{it}-\text{k}}+\sum_{\text{k}=0}^{\text{n}}\beta_{4\text{k}}\Delta {\text{TI}}^{+}{}_{\text{it}-\text{k}}+\sum_{\text{k}=0}^{\text{n}}\delta_{5\text{k}}\Delta {\text{TI}}^{-}{}_{\text{it}-\text{k}}\\ +\sum_{\text{k}=0}^{\text{n}}\beta_{6\text{k}}{\text{X}}_{\text{it}-\text{k}}+\omega_{1}\text{Life }{\text{expectancy}}_{\text{it}-1}+\omega_{2}{\text{EE}}^{+}{}_{\text{it}-1}\\ +\;\omega_{3}{\text{EE}}^{-}{}_{\text{it}-1}+\omega_{4}{\text{TI}}^{+}{}_{\text{it}-1}+\omega_{5}{\text{TI}}^{-}{}_{\text{it}-1}\\ +\omega_{6}{\text{X}}_{\text{it}-1}+\varepsilon_{\text{it}} \end{array}$$

This shows that Equation (4) has now transformed into a non-linear panel ARDL-PMG. It is noteworthy that the estimation procedure of this equation is the same as the linear model. Moreover, the same test of co-integration is applicable in the non-linear panel ARDL-PMG, as this is an extension of the linear ARDL. However, this model provides us the luxury of detecting the impact of positive and negative shocks, separately, on the dependent variable as well. In this regard, the questions pertaining to the non-asymmetric and asymmetric causality between human capital investments, technological innovation, and population health are also addressed in this study, primarily by using the teachings of Dumitrescu and Hurlin (36), and Hatemi-j (37) panel causality tests.

## Data

The analysis for this study has been conducted exclusively for BRICS economies that pertain to Brazil, Russia, India, China, and South Africa, and cover the data collection period from 1991 to 2019. The dataset of all variables has been collected from the World Development Indicators (WDI) that are compiled and updated by the World Bank. Moreover, life expectancy has been considered as a dependent variable, while government education expenditure (EE), and technology innovation (TI), as a proxy of patent applicants, are considered to be the independent variables. In addition to this, the GDP per capita (GDP), the average year of schooling (education), and the number of users of the internet (Internet) have been considered as the control variables in the analysis. Moving on, the transformed technology innovation and GDP data have been considered in terms of the natural logarithm. In Table 1, the mean values of the life expectancy, EE, TI, GDP, education, and internet users are 67.2 years, 4.35%, 8.95, 8.39$, 12.3 years, and 20.2%, respectively, while the standard deviations are 5.93 years, 1.52%, 1.93%, 0.93$, 2.06 years, and 23.5%, respectively.

**Table 1:** Variables definition and data descriptive.

**Variables**	**Symbol**	**Variables**	**Mean**	**Std. Dev**.	**Min**	**Max**
Life expectancy at birth	Life expectancy	Life expectancy at birth, total (years)	67.2	5.93	53.4	76.9
Education expenditure	EE	Government expenditure on education, total (% of GDP)	4.35	1.52	1.44	8.59
Technology innovation	TI	Patent applications, residents	8.95	1.93	4.93	14.1
GDP per capita	GDP	GDP per capita (constant 2010 US$)	8.39	0.93	6.36	9.39
Year of schooling	Education	Average year of schooling	12.3	2.06	7.70	15.6
Internet users	Internet	Individuals using the Internet (% of population)	20.2	23.5	0.00	80.8

## Results and Discussion

Before applying any regression techniques, it was necessary to test the stationarity properties of the considered data. For this purpose, the study has used the Levin–Lin–Chu (LLC) test, the Im–Pesaran–Shin (IPS) test, and the Fisher-ADF tests. The statistical outcomes of the LLC test, IPS test, and ADF test have been presented in Table 2. The findings of these tests show that only a few variables are level stationary, and while a further few are stationary at the first difference. The study also opts for the ARDL and NARDL models, in order to investigate the symmetric and asymmetric nexus among the variables in the short-run and long-run, in the panel of BRICS countries. Moreover, Table 3 delivers the findings of the short-run and long-run estimates of the ARDL and NARDL models, along with the outcomes of various diagnostic tests.

**Table 2:** Unit root tests.

	**LLC**			**IPS**			**ADF**		
	**I(0)**	**I(1)**	**Decision**	**I(0)**	**I(1)**	**Decision**	**I(0)**	**I(1)**	**Decision**
Life expectancy	−4.825^***^		I(0)	−3.654^***^		I(0)	−3.434^***^		I(0)
EE	−0.340	−1.925^*^	I(1)	−0.123	−3.546^***^	I(1)	−1.154	−4.435^***^	I(1)
TI	−3.086^***^		I(0)	−1.044	−5.740^***^	I(1)	−0.063	−10.54^***^	I(1)
GDP	−0.531	−2.121^**^	I(1)	−0.704	−3.371^***^	I(1)	1.787	−4.984^***^	I(1)
Education	−1.082	−3.516^***^	I(1)	−0.678	−3.617^***^	I(1)	−1.044	−5.670^***^	I(1)
Internet	−0.160	−4.384^***^	I(1)	−0.684	−2.589^***^	I(1)	−1.162	−2.878^***^	I(1)

*** *p < 0.01*,

** *p < 0.05, and*

* *p < 0.1*.

**Table 3:** Panel ARDL and NARDL PMG estimation.

	**Panel ARDL**				**Panel NARDL**		
**Variable**	**Coefficient**	**Std. error**	***t*****-Stat**	**Variable**	**Coefficient**	**Std. error**	***t*****-Stat**
**Long run**							
EE	3.717^***^	1.080	3.442	EE_POS	0.804^***^	0.101	7.964
TI	0.836	0.730	1.146	EE_NEG	−3.143	0.229	13.71
GDP	4.851^***^	1.051	4.059	TI_POS	2.608^***^	0.336	7.759
Education	8.557^***^	2.598	3.293	TI_NEG	−1.168	0.601	1.943
Internet	0.030	0.024	1.224	GDP	7.747^***^	0.524	14.78
				Education	1.804^***^	0.198	9.097
				Internet	0.075^***^	0.010	7.440
**Short run**							
D (EE)	−0.091	0.130	0.705	D (EE_POS)	0.014	0.049	0.283
D [EE (−1)]	0.007	0.062	0.116	D [EE_POS (−1)]	−0.047	0.124	0.381
D (TI)	0.259^*^	0.155	1.671	D (EE_NEG)	−0.665	0.708	0.940
D [TI (−1)]	−0.312	0.318	0.980	D [EE_NEG (−1)]	0.620	0.432	1.433
D (GDP)	0.399^*^	0.224	1.781	D (TI_POS)	0.530^*^	0.311	1.709
D [GDP (−1)]	0.585	0.854	0.685	D [TI_POS (−1)]	0.204	0.186	1.098
D (Education)	−0.040	0.056	0.715	D (TI_NEG)	−0.010	0.157	0.064
D [Education (−1)]	−0.125	0.082	1.525	D [TI_NEG (−1)]	−0.636^*^	0.365	1.744
D (Internet)	0.008^**^	0.004	2.000	D (GDP)	0.919^*^	0.542	1.695
D [Internet (−1)]	0.004	0.003	1.166	D [GDP (−1)]	−0.032	0.463	0.068
C	−0.339	1.414	0.240	D (Education)	−0.186	0.234	0.793
				D [Education (−1)]	−0.065	0.041	1.608
				D (Internet)	0.008^***^	0.003	2.666
				D [Internet (−1)]	0.004	0.005	0.925
				C	0.062	0.217	0.284
**Diagnostic**							
Log likelihood	369.6^***^			Log likelihood	456.95^***^		
LM	1.458			LM	1.979		
F-test	5.698^***^			*F*-test	6.988^***^		
ECM (−1)	−0.221	0.117	1.888	ECM (−1)	−0.423^**^	0.210	2.014
				Wald-EE-L	5.988^***^		
				Wald-EE-S	1.326		
				Wald-TI-L	7.612^***^		
				Wald-TI-S	0.123		

*** *p < 0.01*,

** *p < 0.05, and*

* *p < 0.1*.

The long-run findings of ARDL show that the government sector education expenditure tends to positively affect the life expectancy in BRICS economies. It also demonstrates that a 1% increase in government sector education expenditures results in increasing the life expectancy by 3.717% in these economies. In addition to this, GDP and education have a significant and positive impact on the life expectancy in BRICS economies, specifically in the long-run. It shows that due to a 1% upsurge in the GDP per capita and education, the life expectancy increases by 4.851 and 8.557% in the BRICS countries. However, it is noteworthy that technological innovation and the number of internet users have no effect on the level of life expectancy in BRICS economies, as shown by a statistically insignificant coefficient estimate of both variables. The short-run findings of ARDL show that technological innovation, GDP per capita, and internet users positively affect the life expectancy index in BRICS economies. However, the impact of the government sector education expenditure and education on the general life expectancy is statistically insignificant in the short-run. For diagnostic testing, a certain number of tests have also been applied. These include the log-likelihood test, LM test, *F*-test, and the ECM test. The significant coefficient estimate of the log likelihood test confirms the goodness of fit of model. Moreover, the statistically significant coefficient estimates of F-statistics and ECM confirm the existence of the long-run cointegration among the variables. Other than that, the ECM term holds a negative sign with a value 0.221, which states that almost 22% convergence toward the equilibrium level is likely to occur in period of one year. Moreover, the coefficient estimate of LM shows there is no issue of serial correlation in the data that has been taken into account.

The long-run outcomes of the NARDL show that the positive component of the government sector education expenditure has a significant and positive impact on the life expectancy in BRICS economies. It reveals that in response to a 1% increase in the positive component of the government sector education expenditure, life expectancy in BRICS economies increases up to 0.804%. On the other hand, the negative shocks in the government sector education expenditure result in decreasing life expectancy in BRICS economies in the long-run. In more precise terms, a 1% decrease in the negative component of the government sector education expenditure leads to a reduction in the life expectancy by 3.143% in the BRICS economies. The positive shocks in technological innovations tend to have a significant positive impact on the life expectancy in the long-run, which propagates that a 1% upsurge in technological innovation leads to a 2.608% increase in the life expectancy in the BRICS economies. Conversely, the negative shocks in technological innovation negatively affect the life expectancy, as a 1% decrease in the negative shocks in technological innovation result in a 1.168% reduction in the life expectancy in BRICS economies, specifically in the long-run.

These findings are also consistent with Oster et al. (38), who noted that human capital investment is one of the key inputs of health outcomes. The results revealed that human capital investment raises awareness and information about good health, in return, the human capital improves the level of life expectancy. Moreover, the human capital theory predicts a longer life expectancy, primarily because human capital is a key input of the health outcomes. In addition to this, these findings are also supported by Manton et al. (39), who noted that technology innovation is more responsive to health care efficiency. The average life expectancy of humans can increase with improvements in education, affordable housing, sanitation, and the effective advancements in medical treatments. In this context, it is observed that the progress in the technological sector permits everyone to improve his/her health individually, which helps in improving the life expectancy on an individual basis. In many countries, the level and percentage of life expectancy has reached up to seventy years and above. These significant gains are achieved due to better healthcare services and facilities, better nutrition, improved public health, and, most significantly, due to the application of technological innovations. The growth in technological innovations benefits longevity and healthy aging, primarily by empowering people to spend gratifying and healthier lives at all age groups. Technological innovations contribute in several ways when keeping people more physically active, and enabling them to spend an independent living style. For instance, by adopting smart home technology, the detection and management of disease conditions at the initial stages, continuous involvements in the workforce, and maintaining social relations by dropping social isolation, etc., the contribution of technological innovations can be fathomed. In order to ensure the maximum benefits of technological innovations on longevity and aging, there is also a need to design such inclusive technologies that benefit every one of the direct and indirect stakeholders.

As far as the findings of the other control variables are concerned, all three variables (i.e., GDP, education, and internet) have a significant and positive impact on the life expectancy in BRICS economies, particularly in the long run. In this regard, due to a 1% increase in the GDP, education, and internet users, the life expectancy increases by 7.747, 1.804, and 0.075%, respectively, in the long run. The short-run outcomes of NARDL also show that only the positive component of technological innovation has a significant and positive impact on the life expectancy. In the case of the control variables that are taken into account, the GDP per capita and the internet users, positively and significantly, influence the life expectancy in BRICS economies.

Moving on, the findings of the diagnostic tests reveal that the log-likelihood test result is statistically significant, confirming the goodness of fit of the model. Moreover, the F-statistics and the ECM results are statistically significant, which confirms and validates the existence of the element of long-run cointegration among the considered variables. It must also be noted that the ECM term is observed to be negative, with a value of 0.423, which shows that an almost 42% convergence toward equilibrium will occur in a time span of 1 year. The coefficient estimate of the LM also confirms that there was issue of the serial correlation in the data. Other than this, the Wald test confirms the presence of an asymmetric relationship between the government sector education expenditures, technological innovation, and the life expectancy in BRICS economies in the long-run. However, the Wald test does not establish any asymmetry between these variables in the short run.

Table 4 reported the non-symmetric and asymmetric causality relationship among the concerned variables for BRICS economies. In this regard, the non-asymmetric causality analysis outcomes for BRICS indicate that there exists a bidirectional causality from the education expenditure to the life expectancy, and technology innovation to the life expectancy. However, the asymmetric causality test findings confirm the bidirectional causality relationship in the positive and negative shocks of the education expenditure, to the life expectancy for BRICS. These revelations imply that an increase and decrease in human capital investment is affected by life expectancy, and alternatively, an increase in life expectancy promotes an increase in human capital investment. Our findings also show evidence of bidirectional asymmetric Granger-causal relationship between technology innovation and life expectancy. The shocks of the technology innovation also seem to affect the life expectancy of the population of BRICS economies.

**Table 4:** Symmetric and asymmetric Granger causality.

	**Symmetric**				**Asymmetric**		
**Null hypothesis**	***W*****-Stat**.	**Zbar-Stat**.	**Prob**.	**Null hypothesis**	***W*****-Stat**.	**Zbar-Stat**.	**Prob**.
EE → Life expectancy	25.88^***^	21.72	0.000	EE_POS → Life expectancy	20.07^***^	16.22	0.000
Life expectancy → EE	10.63^***^	7.758	0.000	Life expectancy → EE_POS	9.270^***^	6.425	0.000
TI → Life expectancy	8.840^***^	6.105	0.000	EE_NEG → Life expectancy	25.68^***^	21.33	0.000
Life expectancy → TI	4.505^*^	2.119	0.034	Life expectancy → EE_NEG	5.371^***^	2.877	0.004
GDP → Life expectancy	42.64^***^	37.18	0.000	TI_POS → Life expectancy	23.81^***^	19.60	0.000
Life expectancy → GDP	5.702^***^	3.220	0.001	Life expectancy → TI_POS	4.227^*^	1.835	0.067
EDUCATION → Life expectancy	31.60^***^	27.07	0.000	TI_NEG → Life expectancy	6.686^***^	4.073	0.000
Life expectancy → EDUCATION	7.277^***^	4.667	0.000	Life expectancy → TI_NEG	6.215^***^	3.644	0.000
INTERNET → Life expectancy	20.17^***^	16.57	0.000	GDP → Life expectancy	42.64^***^	37.18	0.000
Life expectancy → INTERNET	7.597^***^	4.962	0.000	Life expectancy → GDP	5.702^***^	3.220	0.001
TI → EE	6.795^***^	4.225	0.000	EDUCATION → Life expectancy	31.60^***^	27.03	0.000
EE → TI	7.125^***^	4.528	0.000	Life expectancy → EDUCATION	7.277^***^	4.667	0.000
GDP → EE	6.099^***^	3.585	0.000	INTERNET → Life expectancy	20.17^***^	16.52	0.000
EE → GDP	4.479^**^	2.095	0.036	Life expectancy → INTERNET	7.597^***^	4.962	0.000
EDUCATION → EE	1.677	0.481	0.631	EE_NEG → EE_POS	3.086	0.797	0.426
EE → EDUCATION	5.819^***^	3.327	0.001	EE_POS → EE_NEG	7.544^***^	4.854	0.000
INTERNET → EE	3.457	1.156	0.248	TI_POS → EE_POS	5.048^**^	2.582	0.010
EE → INTERNET	6.026^***^	3.517	0.000	EE_POS → TI_POS	4.871^**^	2.421	0.016
GDP → TI	5.056^***^	2.626	0.009	TI_NEG → EE_POS	3.822	1.467	0.142
TI → GDP	2.694	0.454	0.650	EE_POS → TI_NEG	3.132	0.839	0.402
EDUCATION → TI	2.314	0.105	0.916	GDP → EE_POS	5.917^***^	3.373	0.001
TI → EDUCATION	4.525^**^	2.138	0.033	EE_POS → GDP	5.190^***^	2.712	0.007
INTERNET → TI	1.704	0.456	0.649	EDUCATION → _POS	1.235	0.888	0.375
TI → INTERNET	7.010^***^	4.422	0.000	EE_POS → EDUCATION	4.746^**^	2.308	0.021
EDUCATION → GDP	4.722^**^	2.319	0.020	INTERNET → EE_POS	3.429	1.109	0.268
GDP → EDUCATION	6.192^***^	3.670	0.000	EE_POS → INTERNET	6.083^***^	3.525	0.000
INTERNET → GDP	1.681	0.477	0.634	TI_POS → EE_NEG	4.281^*^	1.885	0.060
GDP → INTERNET	9.908^***^	7.086	0.000	EE_NEG → TI_POS	4.292^*^	1.895	0.058
INTERNET → cause EDUCATION	5.212^***^	2.769	0.006	TI_NEG → EE_NEG	83.62^***^	75.79	0.000
EDUCATION → INTERNET	9.382^***^	6.603	0.000	EE_NEG → TI_NEG	8.716^***^	5.921	0.000
				GDP → EE_NEG	2.988	0.708	0.479
				EE_NEG → GDP	3.934	1.569	0.117
				EDUCATION → EE_NEG	2.257	0.042	0.966
				EE_NEG → EDUCATION	2.818	0.553	0.580
				INTERNET → EE_NEG	1.468	−0.676	0.499
				EE_NEG → INTERNET	5.372^***^	2.876	0.004
				TI_NEG → TI_POS	13.96^***^	10.65	0.000
				TI_POS → TI_NEG	2.636	0.387	0.699
				GDP → TI_POS	3.198	0.899	0.369
				TI_POS → GDP	3.933	1.568	0.117
				EDUCATION → TI_POS	1.794	−0.379	0.705
				TI_POS → EDUCATION	5.795^***^	3.263	0.001
				INTERNET → TI_POS	1.712	−0.453	0.650
				TI_POS → INTERNET	6.475^***^	3.882	0.000
				GDP → TI_NEG	3.649	1.310	0.190
				TI_NEG → GDP	3.552	1.220	0.222
				EDUCATION → TI_NEG	5.234^***^	2.752	0.006
				TI_NEG → EDUCATION	1.759	−0.411	0.681
				INTERNET → TI_NEG	8.522^***^	5.744	0.000
				TI_NEG → INTERNET	1.849	−0.329	0.742
				EDUCATION → GDP	4.722^**^	2.319	0.020
				GDP → EDUCATION	6.192^***^	3.670	0.000
				INTERNET → GDP	1.681	−0.477	0.634
				GDP → INTERNET	9.908^***^	7.086	0.000
				INTERNET → EDUCATION	5.212^***^	2.769	0.006
				EDUCATION → INTERNET	9.382^***^	6.603	0.000

*** *p < 0.01*,

** *p < 0.05, and*

* *p < 0.1*.

## Conclusion and Policy Implications

The level of global life expectancy has widened by almost 20 years over the past five decades. In this context, researchers have actively been inspecting the key factors that determine the life expectancy and health performance indicators of nations. Thus, this study has been aimed toward identifying the asymmetric impact of human capital investment and technology innovation on the life expectancy in BRICS countries. This study also implements a panel non-linear autoregressive distributed lag (NARDL) model, in order to examine the long-run and short-run dynamics of human capital investment and technology innovation for the health outcomes that are taken into account. Other than that, the study also employs an asymmetric causality test in the context of the variables proposed by (37).

One of the primary purposes of this study was to examine the dynamic impact of human capital investment and technology innovation, on the health outcomes for BRICS countries from 1991 and 2019. The findings of the study revealed an asymmetric impact of human capital investment, and technology innovation on the health of individuals. However, we have found that a positive shock in human capital investment and technology innovation helps to increase life expectancy, while a negative shock in the human capital investment and technology innovation leads to a reduction in life expectancy. Most importantly, a positive technology innovation shock improves the life expectancy in the BRICS in short run. Furthermore, we also find that a negative shock in technology innovation has an insignificant impact on the life expectancy, in the short run. Findings also indicate that positive and negative education expenditure has insignificant short-run asymmetric effects on the life expectancy in BRICS. Moreover, the revelations of the asymmetric causality analysis for BRICS indicate that there exists a bidirectional asymmetric causality from education expenditure to life expectancy, and technology innovation to life expectancy.

When taking into consideration the implications of the study, it is suggested that the government should raise the level of public financing for the education and healthcare sector of the economy. Moreover, the government should more allocate more public funds toward proper research and development. BRICS economies should ideally lay more emphasis on technological innovation in the healthcare sector. Other than that, the government should raise health awareness among people, through smart technology. Governments should also encourage private investment in the technology sector, and work actively toward improving the national health system for better health outcomes. In addition to this, the federal and local governments should promote a healthy lifestyle (exercise, diet, behavior) and protect and preserve the environment (air, soil, water), *via* social media and smart technology.

This study has certain limitations as well. Moreover, this study does not cover the health systems and physical environment of BRICS regions, which are equally important for health efficiency. Further work is called for, in order to examine the relationship between the health systems, physical environment, and the health status of women and men.

## Data Availability Statement

The original contributions presented in the study are included in the article/supplementary material, further inquiries can be directed to the corresponding author.

## Author Contributions

L-PY: conceptualization, software, data curation, and writing—original draft preparation. G-GH: methodology, visualization, investigation, writing—reviewing, and editing. All authors contributed to the article and approved the submitted version.

## Conflict of Interest

The authors declare that the research was conducted in the absence of any commercial or financial relationships that could be construed as a potential conflict of interest.

## References

[1] Bentham J. The Theory of Legislation. London: Kegan Paul (Original work published 1802) (1931).

[2] Becker GS. Investment in human capital: a theoretical analysis. J Polit Econ. (1962) 70:9–49. 10.1086/258724

[3] Schultz TW. Reflections on investment in man. J Polit Econ. (1962) 70:1–8. 10.1086/258723

[4] Mirowsky J Ross CE. Education, personal control, lifestyle and health: a human capital hypothesis. Res Aging. (1998) 20:415–49. 10.1177/0164027598204003

[5] Feldman R Finch M Dowd B Cassou S. The demand for employment-based health insurance plans. J Hum Resour. (1989) 24:115–42. 10.2307/145935

[6] Guralnik JM LaCroix AZ Abbott RD Berkman LF Satterfield S Evans DA Wallace RB. Maintaining mobility in late life. I. Demographic characteristics and chronic conditions. Am J Epidemiol. (1993) 137:845–57. 10.1093/oxfordjournals.aje.a116746 8484376

[7] Kunst AE Mackenbach JP. The size of mortality differences associated with educational level in nine industrialized countries. Am J Public Health. (1994) 84:932–7. 10.2105/AJPH.84.6.932 8203689 PMC1614960

[8] Ross CE Wu C. The links between education and health. Am Soc Rev. (1995) 60:719–45. 10.2307/2096319

[9] Williams DR. Socioeconomic differentials in health: a review and redirection. Soc Psychol Q. (1990) 53:81–99. 10.2307/2786672

[10] Wilkinson CB Spurlock J. The mental health of Black Americans. In: Ethnic Psychiatry. Berlin: Springer. p. 13–60.

[11] Doornbos G Kromhout D. Educational level and mortality in a 32-year follow-up study of 18-year-old men in The Netherlands. Int J Epidemiol. (1990) 19:374–9. 10.1093/ije/19.2.374 2376450

[12] Blackburn JT Bell DR Norcross MF Hudson JD Engstrom LA. Comparison of hamstring neuromechanical properties between healthy males and females and the influence of musculotendinous stiffness. J Electromyogr Kinesiol. (2009) 19:e362–9. 10.1016/j.jelekin.2008.08.005 18829346

[13] Andergassen R Nardini F Ricottilli M. Innovation and growth through local and global interaction. J Econ Dyn Control. (2009) 33:1779–95. 10.1016/j.jedc.2009.04.003

[14] Mansfield E. Contribution of R&D to economic growth in the United States. Science. (1972) 175:477–86. 10.1126/science.175.4021.477 17755645

[15] Nadiri MI. Innovations and Technological Spillovers. NBER working paper. (1993).

[16] Romer PM. Increasing returns and long-run growth. J Polit Econ. (1986) 94:1002–37. 10.1086/261420

[17] Santacreu AM. Innovation, diffusion, and trade: theory and measurement. J Monet Econ. (2015) 75:1–20. 10.1016/j.jmoneco.2015.06.008

[18] Maradana RP Pradhan RP Dash S Gaurav K Jayakumar M Chatterjee D. Does innovation promote economic growth? Evidence from European countries. J Innov Entrepreneurship. (2017) 6:1–23. 10.1186/s13731-016-0061-9

[19] Graff Zivin J Neidell M. Environment, health, and human capital. J Econ Lit. (2013) 51:689–730. 10.1257/jel.51.3.689

[20] Vargas-Prada S Martínez JM Coggon D Delclos G Benavides FG Serra C. Health beliefs, low mood, and somatizing tendency: contribution to incidence and persistence of musculoskeletal pain with and without reported disability. Scand J Work Environ Health. (2013) 39:589–98. 10.5271/sjweh.3377 23955508 PMC3819230

[21] Azam M Khan AQ Ozturk I. The effects of energy on investment, human health, environment and economic growth: empirical evidence from China. Environ Sci Pollut Res. (2019) 26:10816–25. 10.1007/s11356-019-04497-4 30778925

[22] Khan M Roy P Matin I Rabbani M Chowdhury R. An adaptive governance and health system response for the COVID-19 emergency. World Dev. (2021) 137:105213. 10.1016/j.worlddev.2020.105213 33012954 PMC7519722

[23] Mahalik JR Sims JP Di Bianca M. Men's head and heart: health beliefs mediating depression's relationship to heart-healthy behaviors. Psychol Men Masculinities. (2021) 22: 422–6. 10.1037/men0000334

[24] Carducci A Fiore M Azara A Bonaccorsi G Bortoletto M Caggiano G. Environment and health: risk perception and its determinants among Italian university students. Sci Total Environ. (2019) 691:1162–72. 10.1016/j.scitotenv.2019.07.201 31466198

[25] Qiu W Chu C Mao A Wu J. The impacts on health, society, and economy of SARS and H7N9 outbreaks in China: a case comparison study. J Environ Public Health. (2018) 2018:2710185. 10.1155/2018/30050581 PMC6046118

[26] Organization WH. BRICS Health and WHO Country Presence Profile. World Health Organization (2017).

[27] Jakovljevic (Michael) M Ogura S. Health economics at the crossroads of centuries - from the past to the future. Front Public Health. (2016) 4:e00115. 10.3389/fpubh.2016.00115 27376055 PMC4899886

[28] Jakovljevic M Potapchik E Popovich L Barik D Getzen TE. Evolving health expenditure landscape of the BRICS nations and projections to 2025. Health Econ. (2017) 26:844–52. 10.1002/hec.3406 27683202

[29] Romaniuk P Poznańska A Brukało K Holecki T. Health system outcomes in BRICS countries and their association with the economic context. Front Public Health. (2020) 8:e00080. 10.3389/fpubh.2020.00080 32296671 PMC7136407

[30] Marten R McIntyre D Travassos C Shishkin S Longde W Reddy S. An assessment of progress towards universal health coverage in Brazil, Russia, India, China, and South Africa (BRICS). Lancet. (2014) 384:2164–71. 10.1016/S0140-6736(14)60075-1 24793339 PMC7134989

[31] Jakovljevic M Groot W Souliotis K. Editorial: health care financing and affordability in the emerging global markets. Front Public Health. (2016) 4:e00002. 10.3389/fpubh.2016.00002 26835444 PMC4720748

[32] Pesaran MH Shin Y Smith RJ. Testing for the ‘Existence of a Long-run Relationship'. Cambridge: Faculty of Economics, University of Cambridge (1996).

[33] Pesaran MH Shin Y Smith RP. Pooled mean group estimation of dynamic heterogeneous panels. J Am Stat Assoc. (1999) 94:621–34. 10.1080/01621459.1999.10474156

[34] Pesaran MH Shin Y Smith RJ. Bounds testing approaches to the analysis of level relationships. J Appl Econometr. (2001) 16:289–326. 10.1002/jae.616

[35] Shin Y Yu B Greenwood-Nimmo M. Modelling asymmetric cointegration and dynamic multipliers in a nonlinear ARDL framework. In: Festschrift in Honor of Peter Schmidt. New York, NY: Springer (2014). p. 281–314. 10.1007/978-1-4899-8008-3_9

[36] Dumitrescu E-I Hurlin C. Testing for Granger non-causality in heterogeneous panels. Econ Model. (2012) 29:1450–60. 10.1016/j.econmod.2012.02.014

[37] Hatemi-j A. Asymmetric causality tests with an application. Empir Econ. (2012) 43:447–56. 10.1007/s00181-011-0484-x

[38] Oster E Shoulson I Dorsey E. Limited life expectancy, human capital and health investments. Am Econ Rev. (2013) 103:1977–2002. 10.1257/aer.103.5.1977 29533058

[39] Manton KG Gu X Lamb VL. Long-term trends in life expectancy and active life expectancy in the united states. Popul Dev Rev. (2006) 32:81–105. 10.1111/j.1728-4457.2006.00106.x

